# SENP7 Potentiates cGAS Activation by Relieving SUMO-Mediated Inhibition of Cytosolic DNA Sensing

**DOI:** 10.1371/journal.ppat.1006156

**Published:** 2017-01-17

**Authors:** Ye Cui, Huansha Yu, Xin Zheng, Rui Peng, Qiang Wang, Yi Zhou, Rui Wang, Jiehua Wang, Bo Qu, Nan Shen, Qiang Guo, Xing Liu, Chen Wang

**Affiliations:** 1 State Key Laboratory of Cell Biology, CAS Center for Excellence in Molecular Cell Science, Institute of Biochemistry and Cell Biology, Shanghai Institutes for Biological Sciences, Chinese Academy of Sciences, Shanghai, China; 2 Department of Rheumatology, Renji Hospital, School of Medicine, Shanghai Jiao Tong University, Shanghai, China; 3 Program in Cellular and Molecular Medicine, Boston Children’s Hospital and Department of Pediatrics, Harvard Medical School, Boston, Massachusetts, United States of America; 4 State Key Laboratory of Natural Medicines and school of Life Science and Technology, China Pharmaceutical University, 639 Longmian Avenue, Jiangning District, Nanjing, China; University of Southern California, UNITED STATES

## Abstract

Cyclic GMP-AMP (cGAMP) synthase (cGAS, a.k.a. MB21D1), a cytosolic DNA sensor, catalyzes formation of the second messenger 2’3’-cGAMP that activates the stimulator of interferon genes (STING) signaling. How the cGAS activity is modulated remains largely unknown. Here, we demonstrate that sentrin/SUMO-specific protease 7 (SENP7) interacted with and potentiated cGAS activation. The small ubiquitin-like modifier (SUMO) was conjugated onto the lysine residues 335, 372 and 382 of cGAS, which suppressed its DNA-binding, oligomerization and nucleotidyl-transferase activities. SENP7 reversed this inhibition via catalyzing the cGAS de-SUMOylation. Consistently, silencing of SENP7 markedly impaired the IRF3-responsive gene expression induced by cGAS-STING axis. SENP7-knockdown mice were more susceptible to herpes simplex virus 1 (HSV-1) infection. SENP7 was significantly up-regulated in patients with SLE. Our study highlights the temporal modulation of the cGAS activity via dynamic SUMOylation, uncovering a novel mechanism for fine-tuning the STING signaling in innate immunity.

## Author Summary

The Cyclic GMP-AMP (cGAMP) synthase (cGAS, a.k.a. MB21D1) is critical for monitoring the pathogen-derived DNA upon microbial infection. Its activity should be dynamically modulated in case the inadvertent recognition of the aberrant self nucleic acids in cytosol leads to severe autoimmune diseases. Protein posttranslational modifications dynamically shape the strength and duration of the immune signaling pathways. It is intriguing to explore whether SUMOylation could modulate the cGAS-initiated signaling. In this study, we characterized sentrin/SUMO-specific protease 7 (SENP7) to specifically potentiate the cGAS activation. Upon microbial DNA challenge, the small ubiquitin-like modifier (SUMO) was conjugated onto cGAS, which suppressed its DNA-binding, oligomerization and nucleotidyl-transferase activities. SENP7 reversed this inhibition via catalyzing the de-SUMOylation of cGAS. Our study sheds new light on the dynamic function of the SUMOylation in cytosolic DNAs-triggered innate immunity response.

## Introduction

Cytosolic aberrant DNA is generally sensed as a danger signal to alert the host about the presence of invading microbes, which triggers the immediate immune responses to control microbial invasion and induces the subsequent adaptive immunity effective for ultimately eradicating infection. Elucidating the cytosolic-DNA-triggered signaling pathway and the relevant regulatory mechanisms represents a fast evolving field to understand the corresponding innate immunity.

Cyclic GMP-AMP (cGAMP) synthase (cGAS, a.k.a. MB21D1 or C6orf150) was recently characterized as a universal cytosolic-DNA sensor, triggering the immune and inflammatory responses in a DNA-sequence-independent manner [1]. The structures of cGAS-DNA complex have been reported [2,3]. cGAS-deficient cells display profound defects in the production of IFN-β and pro-inflammatory cytokines, when challenged by the DNA virus herpes simplex virus 1 (HSV-1) or by the bacteria *Listeria monocytogenes* or *Mycobacterium tuberculosis* (*Mtb*) [4–8]. cGas-knockout mice were more susceptible to lethal infection after exposure to HSV-1 than wild-type mice [8]. In addition, cGAS is essential for innate immune responses against HIV and other retroviruses via detecting the reverse-transcribed retroviral DNA [9]. Interestingly, cGAS possesses the nucleotidyl-transferase catalytic activity, which could be activated by the dsDNA-induced oligomerization [2,3,10]. cGAS catalyzes from ATP and GTP the synthesis of a non-canonical cyclic dinucleotide c[G(2’,5’)pA(3’,5’)p] (referred to as 2’3’cGAMP) [11–15]. Notably, the 2’3’cGAMP is observed to transfer from the producing cells to the neighboring cells via cell-cell junctions, or to the newly infected cells by virions incorporated with cGAMP [16–18].

As a second messenger, cGAMP directly binds to and activates the ER-resident stimulator of interferon genes (STING), a converging adaptor for all known cytosolic DNA sensors [19]. STING is triggered to dimerize and translocate from the endoplasmic reticulum (ER), through the Golgi apparatus, to perinuclear microsomal compartments. Simultaneously, ER-resident autocrine motility factor receptor (AMFR) and insulin induced gene 1 (INSIG1) protein complex catalyzes K27-linked poly-ubiquitination of STING [20,21]. TBK1 selectively binds to K27-linked poly-ubiquitin chains on STING, thus congregating at perinuclear microsomes in a STING-dependent manner. The DNA-induced assembly of the STING-TBK1 complex is required for the activation of TBK1 and the subsequent phosphorylation and nuclear translocation of the transcriptional factor IRF3, ultimately leading to expression of interferon (IFN) and pro-inflammatory cytokines. However, the STING signaling pathway could adversely promote the autoimmune diseases, including systemic lupus erythematosus (SLE), lupus-like diseases, and Aicardi Goutieres syndrome (AGS), when the aberrant self-DNAs is not properly cleared out and accumulate in the cytosol under pathological conditions [22–24]. It is intriguing to dissect the regulatory mechanisms of the cGAS activity, and to understand how cGAS is spatially and temporally modulated to maintain immune balance.

Protein posttranslational modifications (PTM) are effective means to dynamically shape the strength and duration of signal transductions. Ubiquitin and ubiquitin-like proteins (Ubls) are versatile molecular signatures for orchestrating the appropriate innate immune responses [25]. It remains to explore the regulatory role of SUMOylation in the cGAS-STING signaling pathway. Previous studies identified a family of sentrin/SUMO-specific proteases (SENPs), which catalyze the biochemical reaction of de-SUMOylation and modulate the dynamic equilibrium of SUMOylation. SENP family has six members (SENP1- 3 & SENP5-7), each of which exhibits distinct expression patterns and substrate specificity [26,27]. In this study, we characterized SENP7 to specifically potentiate the cGAS activation. Silencing of SENP7 markedly attenuated the cGAS-mediated induction of antimicrobial genes. SENP7-knockdown mice were more susceptible to HSV-1 infection. Mechanistically, the small ubiquitin-like modifier (SUMO) was conjugated onto the lysine residues 335, 372 and 382 of cGAS, which suppressed its DNA-binding, oligomerization and nucleotidyl-transferase activities. SENP7 reversed this inhibition via catalyzing the de-SUMOylation of cGAS. Our study reveals the novel function of SENP7 in the STING signaling, shedding new light on the regulatory role of SUMOylation in innate immunity.

## Results

### SENP7 promotes cytosolic DNA-triggered STING signaling

**Fig 1:**
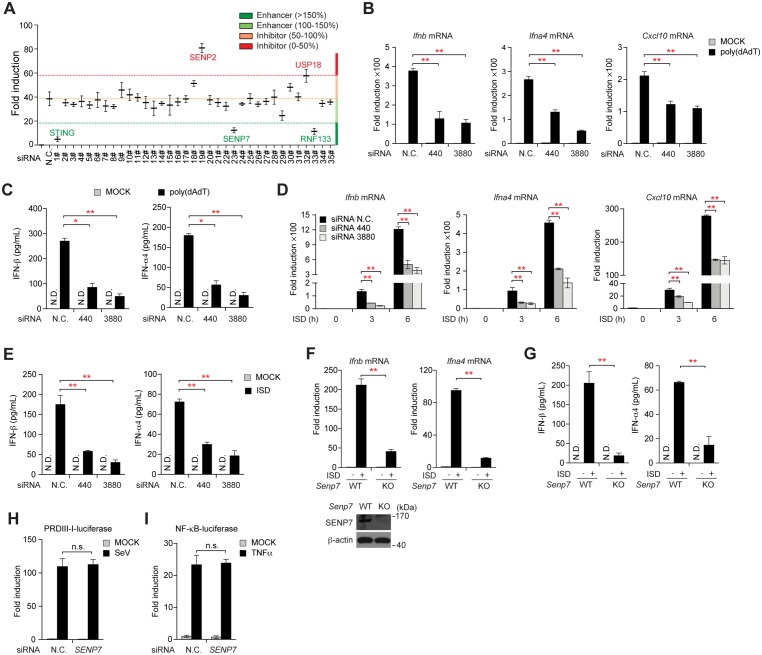
Identification of SENP7 as a new regulator of cGAS-STING signaling. (**A**) IFN-βluciferase and pTK-Renilla reporters were transfected into THP1 cells together with the indicated siRNA. Forty-eight hours after transfection, cells were stimulated with HSV-1 60mer for eight hours before luciferase reporter assays were performed. (**B-E**) MEFs transfected with the indicated siRNAs were stimulated with poly(dA:dT) (**B, C**) or ISD (**D, E**). Induction of *Ifnb*, *Ifna4* and *Cxcl10* mRNAs was measured by quantitative PCR (**B, D**). IFN-β and IFN-α4 production was assayed by ELISA (**C, E**). (**F, G**) *Senp7*^-/-^ MEFs were generated by CRISPR-Cas9-mediated targeting. Induction of *Ifnb* and *Ifna4* mRNAs was measured by quantitative PCR after ISD stimulation (**F, upper panel**). The immunoblots in (**F, lower panel)** show the ablation of SENP7 in *Senp7*^-/-^ MEFs. And the supernatants were collected and assayed for IFN-β and IFN-α4 production by ELISA (**G**). (**H, I**) HEK293 cells were transfected with the nonspecific control (N.C.) or *SENP7* siRNAs together with the indicated reporter plasmids. Forty-eight hours after transfection, the cells were infected with SeV (**H**) or treated with TNF- α (**I**) before luciferase assays were performed. Graphs show the mean ± s.d. and data (B-I) shown are representative of three independent experiments. n.s., not significant; *P < 0.05; **P < 0.01 (two-tailed t-test).

To explore the functions of protein post-translational modifications in STING signaling, we screened a library of small interfering RNAs (siRNAs) that target the E3 ubiquitin ligases, deubiquitinases and SUMO-specific proteases. The 60-mer oligonucleotide dsDNA derived from the HSV-1 genome (referred to as HSV-1 60-mer) was used to stimulate the expression of the IFN-β-luciferase-reporter in the functional assay. As expected, the siRNA against STING dramatically impaired the activation of the IFN-β-luciferase-reporter. Notably, knockdown of *Senp7* or *Rnf133* respectively impaired the same activation to a similar extent. In contrast, knockdown of *Senp2* or *Usp18* displayed a 1.5 fold increase of the luciferase induction (Fig 1A).

It has been established that chronic activation of STING-dependent immune signaling and subsequent type I IFN production by aberrant DNA species play a crucial role in inflammatory disorders such as systemic lupus erythematosus (SLE) [28,29]. To assess the association of the genes fished out from the above screening (*Senp7*, *Rnf133*, *Senp2* and *Usp18*) with SLE, we collected peripheral blood samples from 27 patients with SLE and 28 healthy donors to check the mRNA abundance of these genes. Interestingly, the expression levels of *SENP7* transcripts were highly elevated in the samples of SLE patients than those in healthy donors, while the expression levels of other genes are comparable between SLE patients and healthy donors (S1A Fig). Also, the abundance of *SENP7* mRNA in the samples of SLE patients was positively correlated with that of IFN-inducible genes (S1B Fig), indicating that the expression of SENP7 was positively correlated with IFN production and SLE. These data implied that SENP7 might be important in DNA-triggered STING signaling.

Two different *Senp7* siRNAs (*Senp7* siRNA 440 and *Senp7* siRNA 3880) were further employed to test whether SENP7 regulates the expression of IRF3-responsive genes induced by cytosolic DNA stimuli, as measured by qPCR (quantitative PCR) and ELISA (enzyme-linked immunosorbent assay) (S1C Fig). It was observed that silencing of *Senp7* drastically down-regulated the expression of the IRF3-responsive genes (*Ifnb*, *Ifna4* and *Cxcl10*) in MEFs, stimulated by poly(dA:dT) or ISD (Fig 1B–1E). However, silencing of SENP3 had no such effect (S1D Fig). Furthermore, *Senp7*^-/-^ MEFs were generated by CRISPR-Cas9-mediated targeting (Fig 1F, **lower panel**). Consistently, the absence of Senp7 markedly crippled the cytosolic DNA-triggered antiviral gene expression (Fig 1F and 1G). In contrast, the SeV (Sendai virus)-induced activation of the IFN-β-luciferase reporter was barely affected when silencing *SENP7*, indicating that SENP7 did not regulate the RIG-I/MDA5-mediated signaling (Fig 1H). Silencing of *SENP7* also displayed no effect on TNF- α -induced NF-κB activation (Fig 1I). Collectively, these data suggest that SENP7 potentially promotes the STING signaling.

### SENP7 potentiates STING signaling via catalyzing de-SUMOylation

**Fig 2:**
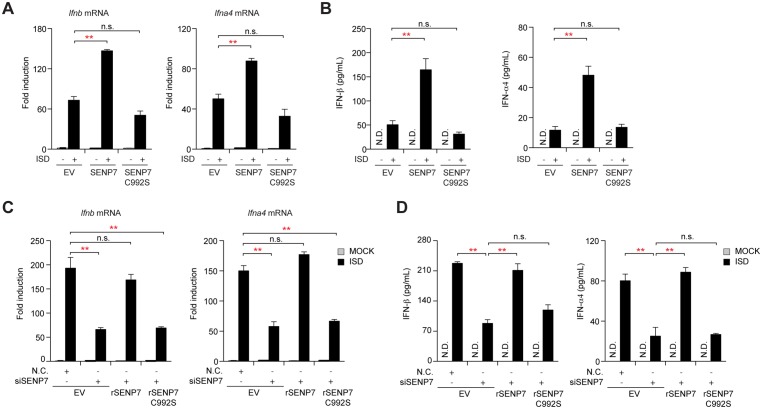
De-SUMOylation activity of SENP7 is essential in potentiating STING signaling. (**A**) MEFs transfect with empty vector (EV) or vector for the expression of Flag-tagged wild-type SENP7 or SENP7 C992S. Induction of *Ifnb* and *Ifna4* mRNAs was measured by quantitative PCR after ISD stimulation. (**B**) MEFs were treated as in (**A**), and the supernatants were collected and assayed for IFN-β and IFN-α4 production by ELISA. (**C**) MEFs were transfected with the nonspecific control (N.C.) or *Senp7* siRNA and then rescued with the indicated siRNA-resistant SENP7 constructs. After ISD stimulation, induction of *Ifnb* and *Ifna4* mRNA was measured by quantitative PCR. (**D**) MEFs were treated as in (**C**), and the supernatants were collected and assayed for IFN-β and IFN-α4 production by ELISA. Graphs show the mean ± s.d. and data shown are representative of three independent experiments. n.s., not significant; **P < 0.01 (two-tailed t-test).

Given that SENP7 is a SUMO-specific protease, we explored whether its regulation of the STING signaling was dependent on its de-SUMOylation activity. We generated the SENP7 C992S mutant (cysteine to serine substitution at position 992), which is deprived of the de-SUMOylation biochemical activity [30]. Ectopic-expression of SENP7 WT potentiates the ISD-triggered STING signaling, as evidenced by higher levels of IFNs transcription and secretion (Fig 2A and 2B). In contrast, exogenous expression of the SENP7 C992S mutant failed to promote the production of IFNs.

Alternatively, ‘rescue’ experiments were performed in *Senp7*-silenced MEFs. We generated two RNA interference (RNAi)-resistant SENP7 constructs, namely rSENP7 WT and rSENP7 C992S, in which silent mutations were introduced into the sequence targeted by the siRNA without changing the amino acid sequence of the corresponding proteins. MEFs were first transfected with control or SENP7 siRNAs, followed by transfection of the control or indicated rSENP7 plasmids, respectively. As shown in Fig 2C and 2D, the attenuation of IFNs production in *Senp7*-silenced cells was relieved by introducing rSENP7 WT. Notably, rSENP7 C992S was unable to rescue the deficiency of the DNA-induced IFNs expression. Consistently, reconstitution of the wild-type SENP7, rather than SENP7 C992S, is able to rescue the deficiency of the DNA-induced IRF3-responisve genes expression in Senp7-/- MEFs (S1E and S1F Fig). Taken together, these results suggest that the de-SUMOylation activity of SENP7 is essential for modulating the STING signaling pathway.

### SENP7 regulates the cytosolic DNA sensor cGAS *per se*

**Fig 3:**
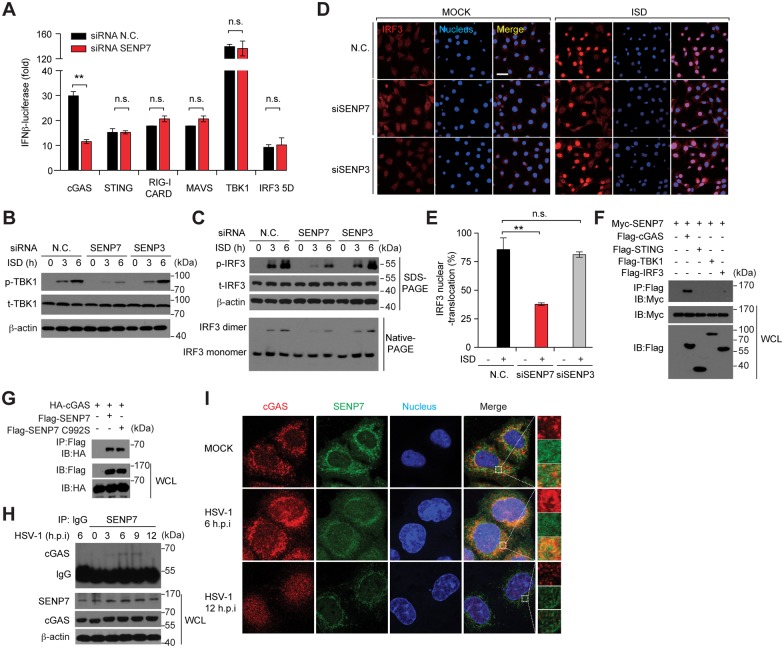
SENP7 regulates STING signaling through direct interaction with cGAS. (**A**) The indicated siRNAs were transfected into HEK293T cells together with IFN-β-luciferase and pTK-Renilla reporter plasmids. 48 hr after transfection, cells were transfected again with cGAS, STING, RIG-I CARD, MAVS, TBK1 or IRF3-5D for sixteen hours before luciferase assays were performed. (**B**) MEFs transfected with the indicated siRNAs were treated or not with ISD for various time periods, and cell extracts were analyzed for TBK1 phosphorylation. (**C**) MEFs transfected with the indicated siRNAs were treated or not with ISD for various time periods, and cell extracts were analyzed for IRF3 phosphorylation and IRF3 dimerization by SDS-PAGE and native PAGE, respectively. (**D, E**) MEFs transfected with the indicated siRNAs were treated with ISD for 4 hours, stained with the antibody against IRF3, and imaged by confocal microscopy (**D**). Cells with nuclear IRF3 staining are counted as a percentage of total cells (n = 100 cells per sample) (**E**). Scale bars represent 50 μm. (**F, G**) HEK293T cells were transfected with the indicated plasmids. 24 hr after transfection, cell lysates were immunoprecipitated with an anti-Flag antibody and then immunoblotted with the indicated antibodies. (**H**) MEFs were infected or not with HSV-1 for the indicated time periods, and the cell lysates were immunoprecipitated with an anti-SENP7 antibody or normal IgG, and then immunoblotted with the indicated antibodies. (**I**) HeLa cells were infected or not with HSV-1 for the indicated time periods, stained with indicated antibodies and imaged by confocal microscopy. Graphs show the mean ± s.d. and data shown are representative of three independent experiments. n.s., not significant; **P < 0.01 (two-tailed t-test).

To identify the potential target of SENP7, we observed that exogenous expression of cGAS could trigger the expression of IFN-β-luciferase reporter, and this activation was markedly crippled when silencing SENP7. In contrast, knockdown of SENP7 did not affect the activation of IFN-β-luciferase reporter, when ectopic-expressing STING, TBK1 or IRF3-5D (Fig 3A). Likewise, SENP7 had no apparent effect on cGAMP-induced activation of the IRF3-responsive genes (S2A Fig). Given the hierarchical relationships among these signaling molecules, we reasoned that SENP7 modulates the STING signaling pathway upstream of STING. In addition, knockdown of SENP7 barely affected the expression of IFN-β-luciferase reporter, stimulated by ectopically expressing the CARD domains of RIG-I or MAVS, indicating that SENP7 did not regulate the RIG-I-mediated signaling (Fig 3A).

To corroborate, knockdown of SENP7 attenuated the ISD-induced phosphorylation of TBK1 (Fig 3B). Consistently, knockdown of SENP7 led to an apparent decrease in the phosphorylation and dimerization of IRF3, when stimulating cells with ISD (Fig 3C). The ISD-induced nuclear translocation of IRF3 was markedly diminished when silencing SENP7 (Fig 3D and 3E). In contrast, knockdown of SENP3 displayed no such inhibitory effects (Fig 3B–3E). Neither knockdown of SENP7 had any effect on either phosphorylation of TBK1 and IRF3 or dimerization of IRF3 when cells were treated with RNA stimulus, emphasizing the specific role of SENP7 in cytosolic DNA-triggered innate immune signaling, but not RNA-induced immune response (S2B Fig).

Co-immunoprecipitation (co-IP) assay revealed that SENP7 specifically associated with cGAS, whereas it did not interact with STING, TBK1 or IRF3 (Figs 3F and S2C). cGAS associated with the SENP7 C992S mutant as well as with SENP7 WT, suggesting that the catalytic domain of SENP7 is dispensable for its association with cGAS (Fig 3G). We then mapped the N-terminal domain of SENP7 (1–300 aa) (S2D Fig) and the middle region of cGAS (240–380 aa) (S2E Fig) to respectively mediate this interaction. The association between SENP7 and cGAS was also confirmed endogenously (Fig 3H).

Notably, we observed the dynamic association between cGAS and SENP7, first strengthened upon HSV-1 challenge and then weakened around 12 hours after HSV-1 infection (Fig 3H). The observation was confirmed by a semi-quantitative immunofluorescence time-course of the endogenous SENP7 and cGAS stimulated by HSV-1 (Fig 3I). Confocal microscopy revealed that cGAS displayed a punctate staining pattern and partially co-localized with SENP7 in the cytoplasm of resting cells. Interestingly, their co-localization was strengthened shortly after HSV-1 infection. Unexpectedly, extended HSV-1 infection led to the nuclear-translocation of cGAS and the obvious spatial separation of SENP7 and cGAS. Taken together, these data establish that SENP7 interacts directly and dynamically with cGAS upon HSV-1 infection.

### SENP7 catalyzes the de-SUMOylation of cGAS

**Fig 4:**
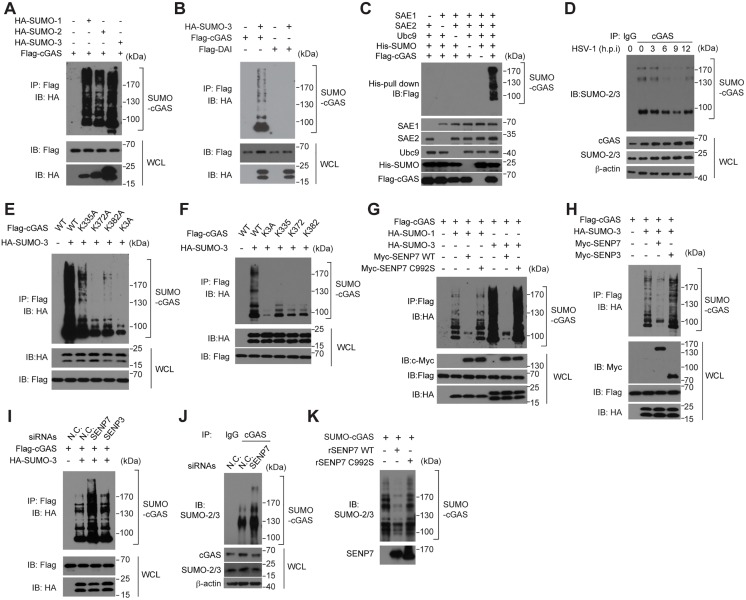
SENP7 modulates the dynamic equilibrium of cGAS SUMOylation. (**A**) Flag-tagged cGAS were transfected into HEK293T cells along with HA-tagged SUMO-1 or HA-tagged SUMO-2/3. 36 hr after transfection, cell lysates were immunoprecipitated with an anti-Flag antibody. The immunoprecipitates were denatured and reimmunoprecipitated with an anti-Flag antibody, and then analyzed by immunoblotting with the indicated antibodies. (**B**) HEK293T cells were transfected with the indicated plasmids. 36 hr after transfection, cell lysates were subjected to a two-step immunoprecipitation and then immunoblotted with the indicated antibodies. (**C**) The SUMOylation reaction mixture contains E1, E2, SUMO and cGAS as indicated. After incubation for 120 min, the mixture was detected by immunoblotting with Flag antibody. (**D**) BMDMs were infected or not with HSV-1 for the indicated time periods, and the lysates were subjected to denaturing immunoprecipitation with an anti-cGAS antibody or normal IgG, and then analyzed by immunoblotting with the indicated antibodies. (**E, F**) Flag-tagged mouse cGAS or its mutants were individually transfected into HEK293T cells along with HA-tagged SUMO-2/3. Cell lysates were subjected to a two-step immunoprecipitation, and then immunoblotted with the indicated antibodies. K3A denotes cGAS with lysine residues 335/ 372/ 382 mutated to alanine. (**G, H**) HEK293T cells were transfected with the indicated plasmids. 36 hr post-transfection, cell lysates were subjected to a two-step immunoprecipitation and then immunoblotted with the indicated antibodies. (**I**) HEK293T cells were transfected with the indicated siRNAs. 36 hr later, Flag-cGAS and HA-SUMO-3 were transfected into the knockdown cells. Cell lysates were subjected to a two-step immunoprecipitation with Flag antibody and immunoblotted with the indicated antibodies. (**J**) MEFs were transfected with the indicated siRNAs and the cell lysates were subjected to denaturing immunoprecipitation with an anti-cGAS antibody or normal IgG, and then analyzed by immunoblotting with the indicated antibodies. (**K**) The deSUMOylation reaction mixture contains SUMOylated cGAS and recombinant WT or catalytically-dead SENP7 as indicated. After incubation for 30 min, the mixture was detected by immunoblotting with the indicated antibodies. All data shown are representative of three independent experiments.

The above observations led us to hypothesize that cGAS could potentially be modified by SUMO. To explore this possibility, we co-expressed Flag-tagged cGAS together with HA-tagged SUMO-1/2/3, respectively. The cell lysates were subjected to the denaturing immunoprecipitation with an anti-Flag antibody. Immunoblot analysis with anti-HA antibody revealed multiple bands that migrated more slowly than cGAS, suggesting that cGAS was robustly modified by SUMO proteins (Fig 4A). In contrast, the cytosolic DNA sensor DAI (a.k.a. DLM-1/ZBP1) was not modified by SUMO (Fig 4B). *In vitro* SUMOylation assay further confirmed that cGAS was bone-fide substrate of SUMO modification, in the presence of recombinant E1 enzyme (SAE1/SAE2) and E2 enzyme (Ubc9) (Fig 4C). In addition, endogenous cGAS was observed to be SUMOylated (Fig 4D). Notably, the level of the cGAS SUMOylation was markedly decreased at the early phase of HSV-1 infection, and gradually restored later, which inversely echoed the pattern of the dynamic interaction between cGAS and SENP7 during HSV-1 infection (Figs 4D, 3H and 3I).

To map the SUMOylation sites on cGAS, we carried out a systematic lysine (K) to alanine (A) mutation scanning. Lysines 335, 372 and 382 were identified to be indispensable for the cGAS SUMOylation, as the SUMOylation of cGAS K335A, K372A or K382A mutants was largely compromised (Figs 4E and S3A). To substantiate, we generated a cGAS (3A) mutant, in which all of the three lysines (335, 372 and 382) were mutated to alanines. As expected, cGAS K3A could barely be modified by SUMO (Fig 4F). On the background of this cGAS K3A mutant, we generated three more cGAS mutants (K335, K372 or K382 respectively), in which only a lysine residue was re-introduced back to the original site. It was observed that the SUMOylation of cGAS reappeared in K335, K372 or K382 cGAS mutants (Fig 4F), indicating that K335, K372 or K382 is also sufficient for cGAS SUMOylation. Consistent with that of cGAS K335A, K372A or K382A mutants, the SUMOylation of cGAS K335R, K372R or K382R mutants (lysine to arginine mutations) was also largely compromised (S3B and S3C Fig). We further performed the sequence alignment of cGAS orthologs and uncovered Lys335, Lys372, Lys382 and their surrounding sequences to be highly conserved across species (S4A Fig). Collectively, these data established that the three lysines (K335, 372 and 382) of cGAS are the major SUMOylation sites on cGAS.

Next, a cell-based de-SUMOylation assay was performed to address whether SENP7 could deconjugate the SUMOylated cGAS. Wild-type SENP7 or catalytically dead SENP7 mutant (SENP7 C992S) was individually co-transfected with cGAS and SUMO. The cell lysates were subjected to immunoprecipitation of Flag-tagged cGAS and then the precipitates were probed for the SUMOylation signal. As expected, cGAS was robustly SUMOylated in the presence of SUMO-1 or SUMO-3 (Fig 4G). Notably, this modification was drastically reduced when expressing SENP7 (Fig 4G). In contrast, the catalytically inactive SENP7 C992S as well as wild-type SENP3 could not influence the SUMOylation status of cGAS (Fig 4G and 4H). Furthermore, knocking down endogenous SENP7 could enhance the basal SUMOylation of Flag-cGAS, whereas knocking down endogenous SENP3 displayed no such effect (Fig 4I). SENP7 knockdown apparently potentiated the SUMOylation of the endogenous cGAS (Fig 4J). *In vitro* assay confirmed that wild-type SENP7, but not SENP7 C992S, could remove the SUMO moiety from the SUMOylated cGAS (Fig 4K). Collectively, these data demonstrate that SENP7 catalyzes the de-SUMOylation of cGAS.

### The SUMOylation of cGAS impairs its activation

**Fig 5:**
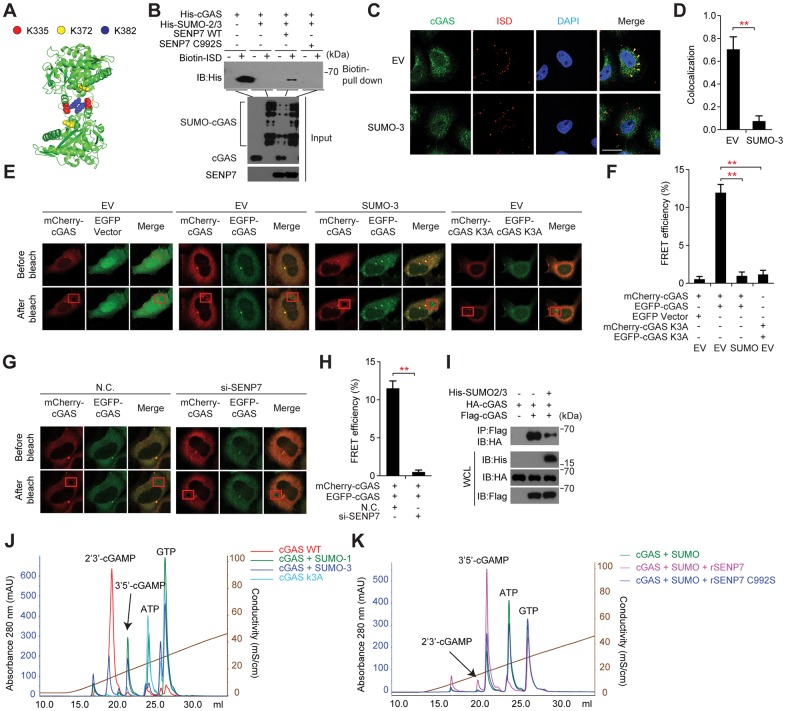
The SUMOylation of cGAS impairs its activation. (**A**) Modeled structure of the cGAS homo-oligomer; red, yellow and blue spheres indicate SUMOylation sites. (**B**) DNA-precipitation assay of cGAS alone or cGAS pre-modified with SUMO by incubating with SUMOylation reaction mixture containing E1, E2 and SUMO or de-SUMOylated cGAS by incubating with SENP7 and SENP7 C992S. (**C, D**) Confocal microscopy of HeLa cells transfected with cyanine dye-3 (Cy3) conjugated ISD (**C**), and co-localization of cGAS with the labeled ISD (**C, D**) (Pearson's correlation coefficient; **D**). Arrows indicate representative co-localization between ISD and cGAS. (**E, F**) N-terminal EGFP-tagged cGAS and C-terminal mcherry-tagged cGAS or cGAS K3A mutant plasmids were transfected into HeLa cells together with or without His-tagged SUMO. cGAS self-association was examined by FRET assay. Rectangle in red (bottom right image of each group) indicates photobleached area (**E**). (**F**), summary of results, presented relative to initial FRET. (**G, H**) The indicated siRNAs were transfected into HeLa cells. 48 hr later, cells were transfected again with N-terminal GFP-tagged cGAS and C-terminal mcherry-tagged cGAS plasmids together with or without His-tagged SUMO. cGAS self-association was examined by FRET assay. Rectangle in red (bottom right image of each group) indicates photobleached area (**G**). (**H**), summary of results, presented relative to initial FRET. (**I**) Cell lysates from HEK293T cells transfected with the indicated plasmids were immunoprecipitated with an anti-Flag antibody, and then immunoblotted with the indicated antibodies. (**J, K**) Reaction products from cGAS, SUMOylated cGAS and cGAS K3A (**J**) as well as WT or catalytically-dead SENP7-treated SUMOylated cGAS (**K**) were analyzed by ion exchange chromatography. 0.2 mg/mL Salmon sperm DNA was added to stimulate cGAS catalytic activity. Graphs show the mean ± s. d. and data shown are representative of three independent experiments. **P < 0.01 (two-tailed t-test). Scale bars, 25 μm.

cGAS functions as a cytosolic DNA sensor by binding to dsDNA and catalyzing the synthesis of the second messenger 2’3’cGAMP. Biophysical and biochemical analysis revealed that the nucleotidyl-transferase (NTase) catalytic activity of cGAS is activated by the dsDNA-induced oligomerization of cGAS. According to the structural data of cGAS and cGAS/dsDNA, we noticed that the SUMOylation sites on cGAS (Lys335, Lys372 and Lys382) are located in either DNA-binding surfaces or dimerization interface of cGAS (Fig 5A). Consistent with previous reports, mutating the three lysines deprived cGAS of its affinity to DNA as well as its catalytic activity (S4B and S4C Fig).

To explore the functional consequence of the cGAS SUMOylation, we expressed and purified cGAS or SUMOylated cGAS, and checked their affinity to the biotin-labeled ISD by pull-down assay with streptavidin-conjugated beads (Fig 5B). Strikingly, the biotin-labeled ISD failed to pull down the SUMOylated cGAS, whereas it could pull down the unmodified cGAS (Fig 5B), indicating that the cGAS SUMOylation impaired its DNA-binding ability. Moreover, as shown in Fig 5B, SENP7 rather than SENP7 C992S could reverse the effect of SUMOylation and restore the binding of cGAS with Biotin-ISD. Consistently, ectopically expressing SUMO abrogated the co-localization of cGAS with ISD (Fig 5C and 5D).

We next performed the fluorescence resonance energy transfer (FRET) assay to explore the cGAS self-association, using green fluorescent protein (GFP)- and mCherry- tagged cGAS. It was observed that there was significant FRET between GFP-cGAS and mCherry-cGAS (~12%), an index for cGAS homo-oligomerization (Fig 5E–5H). However, cells expressing SUMO or silencing SENP7 displayed a significantly lower FRET signal (Fig 5E–5H). Consistently, cells expressing cGAS K3A exhibited a weak FRET signal (Fig 5E and 5F). In addition, immuno-precipitation experiments substantiated that the Flag-tagged cGAS could interact with its HA-tagged counterpart, whereas expressing SUMO crippled this interaction (Fig 5I).

To assess the effect of cGAS SUMOylation on its NTase activity, we conducted the standard enzymatic assay by stimulating cGAS with salmon sperm DNA, followed by ion exchange chromatography. The purified cGAS was subjected to the *in vitro* SUMOylation reaction and enrichment. As expected, cGAS without SUMOylation could efficiently catalyze the synthesis of 2’3’-cGAMP in the presence of ATP, GTP, and salmon sperm DNA (Fig 5J). In contrast, cGAS enriched from the reaction in the presence of SUMO modification system synthesized much less 2’3’-cGAMP, indicating that cGAS catalytic activity was inhibited by the SUMOylation (Fig 5J). Importantly, this inhibition was reversed by SENP7, but not by the catalytic-dead SENP7 (C992S) (Fig 5K). Collectively, these data uncovered the inhibitory function of the SUMOylation for cGAS activation, which is reversed specifically by the de-SUMOylation enzyme SENP7.

### SENP7 restricts HSV-1 and *Listeria monocytogenes* infections *in vitro*

**Fig 6:**
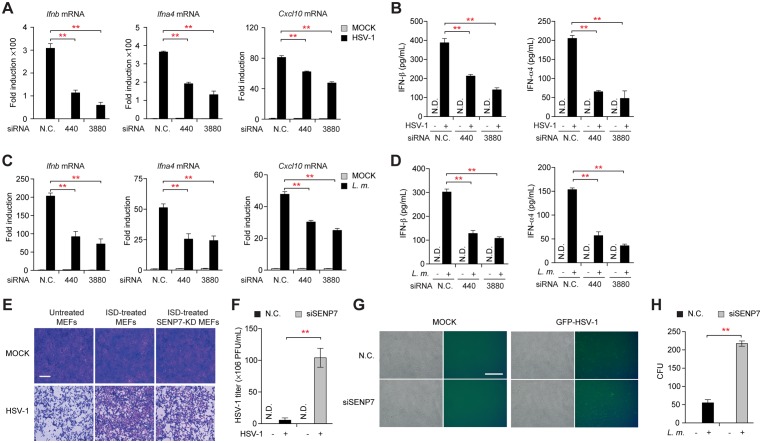
SENP7 modulates innate immune defense against HSV-1 and Listeria monocytogenes invasion. (**A-D**) MEFs transfected with the indicated siRNAs were infected with HSV-1 (**A, B**) or *Listeria monocytogenes* (**C, D**). Induction of *Ifnb*, *Ifna4* and *Cxcl10* mRNAs was measured by quantitative PCR (**A, C**). IFN-β and IFN- α 4 production was assayed by ELISA (**B, D**). (**E**) MEFs transfected with the nonspecific control (N.C.) or *SENP7* siRNAs were treated or not with ISD. Equal volumes of culture supernatants from these treatments were applied to fresh MEF cells, followed by HSV-1 infection. The proliferation of cells was examined by crystal violet staining. Scale bars represent 200 μm. (**F**) MEFs transfected with the nonspecific control (N.C.) or *SENP7* siRNAs were infected with HSV-1. The titers of HSV-1 were determined by standard plaque assay. (**G**) HSV-1-GFP replication in MEFs transfected with the indicated siRNAs was visualized by fluorescence microscopy. (**H**) MEFs transfected with the nonspecific control (N.C.) or *SENP7* siRNAs were infected with *Listeria monocytogenes*. Counts of intracellular bacteria were determined by CFU assay. Graphs show the mean ± s.d. and data shown are representative of three independent experiments. **P < 0.01 (two-tailed t-test).

Robust inductions of IFN-β and interferon-stimulated genes represent one of the immediate responses to the microbial infections. qPCR and ELISA assays revealed that silencing of endogenous SENP7 markedly impaired the production of IFN-β, IFN-α4 or CXCL10, when infecting cells with HSV-1 (Figs 6A, 6B and S5) or *Listeria monocytogenes* (Fig 6C and 6D). Since IFN-β protects host cells against viruses, we further assessed if SENP7 could restrict HSV-1 infection. MEFs were respectively pretreated with culture supernatants from ISD-stimulated *Senp7*-silenced MEFs or control MEFs, followed by HSV-1 infection. Fresh cells pretreated with culture supernatants from *Senp7*-silenced MEFs were more sensitive to HSV-1 infection (Fig 6E).

Next, *Senp7*-silenced MEFs or control MEFs were challenged by HSV-1, and then the titers of HSV-1 were analyzed by standard plaque assay. As shown in Fig 6F, SENP7 knockdown resulted in a 20-fold increase in virus titer as compared with controls. Consistently, knockdown of SENP7 increased the number of HSV-1-GFP-positive cells (Fig 6G). Unlike HSV-1, knockdown of SENP7 has no effect on RNA virus infection, suggesting that SENP7 plays an essential role in restricting DNA virus infection (S6 Fig). In addition, knockdown of SENP7 markedly enhanced the replication of *Listeria monocytogenes* (Fig 6H). These data indicate that SENP7 is important for the host antimicrobial responses.

### SENP7 is essential for the host defense against HSV-1 infection

**Fig 7:**
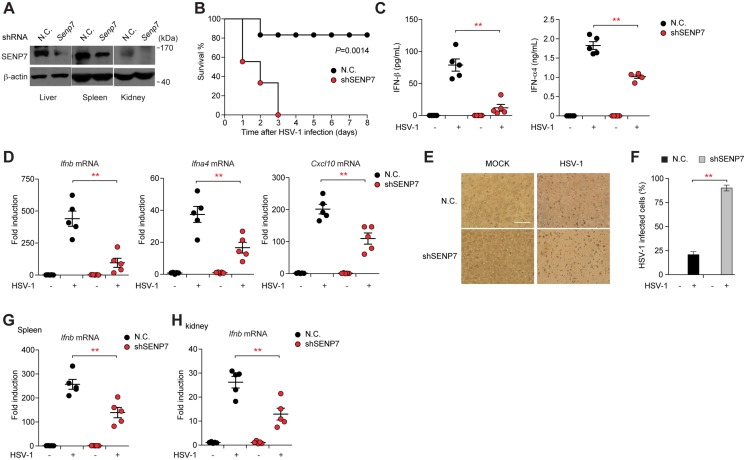
SENP7 is indispensable for the innate defense against HSV-1 infection. (**A**) Immunoblot analysis of SENP7 in lysates of liver, kidney and spleen from mice at 48 hr after transfection with *Senp7* or nonspecific (N.C.) shRNA. (**B**) Survival of mice (n = 9 per group) transfected with *Senp7* or N.C. shRNA and 48 hr later injected intravenously with HSV-1. P = 0.0014 (Mantel-Cox test). (**C**) ELISA of IFN-β and IFN-α4 in serum from mice (n = 5 per group) transfected with *Senp7* or N.C. shRNA and 48 hr later injected intravenously with HSV-1, assessed 6 hr after HSV-1 infection. (**D**) Quantitative PCR of relative *Ifn-b*, *Ifn-a4* and *Cxcl10* mRNA in livers from mice (n = 5 per group) transfected with *Senp7* or N.C. shRNA and 48 hr later injected intravenously with HSV-1, assessed 6 hr after HSV-1 injection. (**E, F**) Brains of the HSV-1-infected mice (n = 6 each) were harvested for immunohistochemistry (IHC) analysis by an anti-HSV-1 antibody. Tissue sections were visualized by microscopy (**E**). Percentages of cells infected by HSV-1 were quantified (**F**). (**G, H**) Quantitative PCR of relative *Ifn-b* mRNA in spleen (**G**) or kindey (**H**) from mice (n = 5 per group) transfected with *Senp7* or N.C. shRNA and 48 hr later injected intravenously with HSV-1, assessed 6 hr after HSV-1 injection. Graphs show the mean ± s.d. and data shown are representative of at least two experiments. *P < 0.05, **P < 0.01 (two-tailed t-test). Scale bars, 50 μm.

Finally, we employed the HSV-1 infection model to investigate the *in vivo* function of SENP7 in innate immunity. First, the endogenous SENP7 was knocked down in mice, via tail vein injection of the SENP7-specific shRNA or control shRNA. The efficiency of the *in vivo* ‘knockdown’ was confirmed, and the depletion occurred most efficiently in the liver, kidney and spleen (Fig 7A). Next, mice were injected intravenously with HSV-1 (the sub-lethal dose), and their survival rates were monitored. As expected, SENP7 knockdown mice (‘SENP7-KD mice’) were more susceptible to HSV-1 than mock knockdown mice (‘control mice’) (Fig 7B). All the SENP7-KD mice died within 3 days, whereas 83% of the infected control mice remained alive until 8 days after HSV-1 infection (Fig 7B). Moreover, the IFN production of the mice was examined *in vivo*. Notably, the SENP7-KD mice displayed a more severe defect in the production of IFN-β and IFN-α4 in sera upon HSV-1 invasion, as compared with the infected control mice (Fig 7C). The expression of *Ifnb*, *Ifna4* and *Cxcl10* mRNAs was significantly lower in SENP7-KD liver tissues (Fig 7D). Consistently, HSV-1 virions were significantly more abundant in the brains of the SENP7-KD mice than those in control mice (Fig 7E and 7F). In addition, the expression of *Ifnb* was also decreased in spleen or kidney tissues of SENP7-KD mice, compared to the control mice (Fig 7G and 7H). Collectively, these data indicate that SENP7 is indispensable for protecting mice against HSV-1 infection.

## Discussion

Recent breakthroughs have uncovered the essential function of the cGAS-STING axis in monitoring cytosolic microbial DNAs, which triggers the expression of type-I interferons (IFNs) and pro-inflammatory cytokines. Given that cGAS senses DNAs in a sequence-independent manner, any aberrant activation of the cGAS by cytosolic DNAs contributes to the pathogenesis of chronic inflammation and autoimmune diseases. Hypothetically, the strength and duration of the cGAS-STING signaling is subjected to multiple layers of stringent modulations, so that the balance of the immune homeostasis could be appropriately maintained. It remains intriguing how cGAS is spatially and temporally modulated in immunity.

In this study, we characterized the SUMO-specific protease SENP7 to potentiate cGAS activation via controlling its dynamic SUMOylation in immune responses. First, silencing of the endogenous SENP7 markedly attenuated the DNAs-triggered expressions of IFNs and ISGs; and this effect could be rescued by exogenously expressing siRNA-resistant SENP7 (rSENP7), but not by the catalytically dead rSENP7 (C992S) mutant. Second, silencing of SENP7 crippled the antimicrobial responses against HSV-1 and *Listeria monocytogene* in host cells. Third, the phosphorylation, dimerization and nuclear-translocation of IRF3 induced by the cytosolic DNAs were markedly impaired when silencing the endogenous SENP7. Fourth, *in vivo* ‘knockdown’ of SENP7 made the mice more permissive to the HSV-1 infection and accelerated the death rate of the infected mice, due to the decreased production of interferons and cytokines. Fifth, the anti-microbial function of SENP7 depended on its de-SUMOylation catalytic activity.

Mechanistically, we observed that cGAS could be modified by SUMO *in vitro* and *in vivo*, whereas another cytosolic DNA sensor DAI could not. This modification was further mapped onto the lysine residues 335, 372 and 382 of cGAS. To substantiate, this SUMOylation markedly impaired the DNA-binding, oligomerization and nucleotidyl-transferase activities of cGAS, suggesting that the SUMO-moiety (~12 kDa) might directly mask the DNA-binding surfaces or oligomerization interfaces. The X-ray crystal structure of the cGAS-dsDNA complex revealed that cGAS interacts with dsDNA through two binding sites (site A and site B) [2]. The DNA-binding triggers cGAS conformational changes to facilitate the subsequent formation of dimers and/or higher oligomers of cGAS through the dimer interface. Notably, the lysine residues 335 and 372 are located respectively in the site B and site A, while the lysine residue 382 is important for the cGAS dimerization. Our cell-based binding assays and functional assays further confirmed the importance of these residues for the cGAS activation. We speculated that the SUMOylation probably creates the steric hindrances for the cGAS recognition of DNAs and/or the cGAS dimerization. Another possibility is that the SUMOylation triggers a corresponding conformational change of cGAS, which influences the charge distribution of cGAS and abolishes the DNA access to cGAS. Future structural analysis of the SUMO-modified cGAS will hopefully provide insights to the specific mechanism.

During revising this manuscript, Hu et al reported that SENP2 deactivates cGAS-STING signaling via modulating the equilibrium of cGAS and STING SUMOylation [31]. Notably, there are some discrepancies between the Hu et al’s and our studies concerning distinct SUMOylation pattern and the role of dynamic SUMOylation in cytosolic DNA sensing pathway. In Hu’s paper, SUMOylation study was performed in the presence of E3 ligase TRIM38, whereas our study was carried out without specific E3 ligases. E3 ligase TRIM38 may catalyze and guide the SUMOylation reaction of cGAS in favor of SUMO-1 modification and covalently linking to specific lysine residues. It is intriguing to screen for potential other E3 ligases catalyzing cGAS SUMOylation and study the spatial and temporal modulations of cGAS activity by the different ligases-mediated SUMOylation. Additionally, it is notable that SENP2 not only possesses the deSUMOylation activity to remove SUMO moiety from cGAS, STING and IRF3, but also involves in processing newly synthesized SUMOs into their mature forms [32]. Thus, it’s unknown whether SENP2 affects cGAS signaling in a direct or indirect manner. In contrast, our study showed SENP7 specifically targets cGAS to regulate the DNA-sensing pathway.

In general, SUMO modification is highly dynamic that is potentially reversed by a family of the deconjugating enzymes (SENP1-3 & SENP5-7). SENPs are emerging as the versatile modulators for orchestrating innate and adaptive immune responses. SENP1 and SENP2 have been reported to regulate the transcriptional activities of IRF8 and IRF3, respectively [33,34]. SENP1 also modulates the T cell synapse organization and T cell activation via controlling the sumoylation status of the kinase PKC-θ [35]. Recently, we characterized SENP6 to attenuate the activation of the inhibitor of NF-κB (IκB) kinase (IKK) via targeting NEMO, thus dampening TLR-induced inflammation [36]. The physiological functions of other SENPs remain largely unknown. In the current study, SENP7 was demonstrated to selectively interact with cGAS and catalyze the de-SUMOylation of cGAS. In contrast, SENP3 did not bind to cGAS or affect the cGAS SUMOylation. Furthermore, SENP3 influenced neither the STING signaling pathway nor the corresponding induction of IFNs and ISGs, highlighting the specific function of SENP7 in cGAS-STING axis. Interestingly, we observed that the interaction between cGAS and SENP7 was transient and dynamic, first increased to a maximum upon HSV-1 challenge and then dropped down after a while. This is consistent with our observation that the level of the SUMOylated cGAS inversely oscillated upon HSV-1 infection, revealing the vital role of SENP7 in fine-tuning the cGAS activity.

The cGAS-STING signaling is recently established as instrumental for the onset and progression of autoimmune diseases [24,37–39]. Interestingly, we observed that SENP7 was significantly up-regulated in patients with autoimmune disease, indicating that SENP7 might synergize the action of cGAS in the pathogenesis of certain autoimmune disorders. The cGAS-STING signaling is also indispensable for the immune sensing of the immunogenic tumors, which ultimately induce the IFN-β production and activate dendritic cells, thus promoting the cross-priming of CD8^+^ T cells against tumor *in vivo* [40–42]. We speculate that SENP7 might modulate the tumor-related immune and inflammatory responses. Future investigation will focus on generating the SENP7 conditional knockout mice and analyzing its potential immunoregulatory function in autoimmunity and cancer immunology.

Taken together, the current study demonstrated that the DNA sensor cGAS is dynamically modified by SUMO. This modification dampens the cGAS activation via suppressing its DNA-binding, oligomerization and nucleotidyl-transferase activities. To counterbalance, SENP7 relieves this inhibition by removing SUMO from cGAS. As an analogy, several recent studies demonstrated the delicate regulations of the adaptor protein STING [43]. For examples, STING is modified by K11, K27 and K48 poly-ubiquitin chains in different contexts [20,44,45]. These poly-ubiquitinations are catalyzed by different ubiquitin E3 ligases and regulate a diverse aspects of the STING action. In addition, STING is phosphorylated by ULK and TBK1, displaying antagonistic functional consequences [46,47]. It is currently unknown about the functional link between ubiquitination and phosphorylation of STING. We assume, a priori, that a more intricate network of the post-translational modifications of cGAS will appear in the coming years to deepen our understanding on how cGAS is fine-tuned or mis-modulated in physiological and pathological conditions.

## Materials and Methods

### Ethics statement

The mice were maintained under specific pathogen-free (SPF) conditions at the Shanghai Institute of Biochemistry and Cell Biology. Animal experiments were carried out in strict accordance with the regulations in the Guide for the Care and Use of Laboratory Animals issued by the Ministry of Science and Technology of the People's Republic of China. The protocol was approved by the Institutional Animal Care and Use Committee of the Shanghai Institute of Biochemistry and Cell Biology, Chinese Academy of Sciences (Permit Number: IBCB0027 Rev2). The study of human peripheral blood samples was approved by the Research Ethics Board of Renji Hospital, Shanghai Jiao Tong University School of Medicine. All adult subjects provided informed consent, and a parent or guardian of any child participant provided informed consent on their behalf. Written informed consent was obtained before sample collection.

### *In vivo* Knockdown of *Senp7*

The plasmid encoding shRNA was delivered into C57BL/6 mice with cationic liposomes PEI transfection reagent (sigma) according to the manufacturer’s instructions [48,49]. The *Senp7*-specific shRNA (or control shRNA; 500 nM) and PEI was each diluted into 100 μl of 5% glucose, then mixed and incubated for 15 min at room temperature at a final N/P ratio of 8. Finally, the mixture (200 μl) was injected into each mouse via tail vein.

### Clinical samples

RNA derived from the PBMCs of patients with SLE, obtained from the Department of Rheumatology at Renji Hospital (Shanghai, China), was used to quantify the gene expression of *SENP7* and IFN-inducible genes. All patients met at least 4 of the American College of Rheumatology revised criteria for the classification of SLE [50]. Individuals with active virus infection were excluded. The patient group composed of 1 man and 26 women, the medium age was 29 years, range from 14 to 70. RNA from the PBMCs of age and gender matched healthy volunteers were used as control.

Peripheral blood samples (5 ml) were collected from each subject and stored in tubes containing EDTA. PBMCs were isolated by Ficoll-Paque PLUS (GE Healthcare) gradient centrifugation and the RNA was extracted using TRIzol reagent according to the manufacture’s instruction.

### Calculation of IFN scores

IFN scores were calculated as described in previous studies [51,52]. Briefly, the mean and standard deviation (SD) for the expression level of each IFN-inducible genes Gene (i) in the healthy donor (HD) group (mean Gene (i)_HD_ and SD Gene (i)_HD_, respectively) were used to obtain a standardized expression level (ST) of each gene for each SLE patient. Then, the standardized expression levels for each patient were summed to obtain IFN score: $\sum_{\left(i=1\right)}^{5}\text{ST}[\text{Gene}(\text{i})]=[\text{Gene}{\left(\text{i}\right)}_{\text{SLE}}-\text{mean Gene}{\left(\text{i}\right)}_{\text{HD}}]/\text{SD}$ Gene (i)_HD_; where i = each of the 3 IFN-inducible genes (*ISG15*, *MX1*, *OAS1*), Gene (i)_SLE_ is the expression level of a particular gene in a given SLE patient.

### Cell culture and transfection

HEK293T (ATCC), MEF (ATCC), L929 (ATCC) and HEK293 (ATCC) cells were cultured using DMEM (Invitrogen) plus 10% FBS (Gibco), supplemented with 1% penicillin-streptomycin (Invitrogen). Vero cells (ATCC) were cultured in MEM (SAFC Biosciences) supplemented with 10% FBS and 1% penicillin-streptomycin. Lipofectamine 2000 (Invitrogen) was used for transient transfection of HEK293T and HEK293 Cells. MEF cells were transfected with X-Gene HP (Roche). Small interference RNA was transfected with Lipofectamine 2000 (Invitrogen) according to the manufacturer’s instructions.

### RNA interference

The siRNA duplexes targeting SENPs were chemically synthesized by Gene-Pharma. The sequences of siRNAs are shown as follows: mouse *Senp7*-440#, 5′- GGA CGA GAA UUC AGA AAG ATT -3′; mouse *Senp7*-3880#, 5′- GAG CUC AUC UGU UCA UAU ATT -3′; human *SENP3*, 5′-GCU UCC GAG UGG CUU AUA ATT-3′; human *SENP7*, 5′-CAA AGU ACC GAG UCG AAU AUU-3′; The nonspecific siRNA (N.C.), 5′-UUC UCC GAA CGU GUC ACG UTT-3′.

### Generation of CRISPR-Cas9 knockout cell line

Human codon-optimized Cas9 (hCas9) and *Senp7*-targeting gRNA-expressing plasmid (pLentiCRISPR) was obtained from GenScript. The target sequence used is 5′-ATC ACC AGC TGA TTT ACA GA-3′. To construct the knockout cell line, MEFs were infected with the lentivirus within which pLentiCRISPR was packaged for two days. Single clones were sorted into a 96-well plate and cultured in the presence of puromycin (8 μ g/mL). On-target CRISPR/Cas9 events were detected by the T7 endonuclease I-cutting assay and the candidate knockout clones were confirmed by sequencing.

### Plasmids and recombinant proteins

SENP7, cGAS, RIG-I, STING, TBK1, IRF3 cDNAs were constructed using standard PCR techniques from the thymus cDNA library and subsequently inserted into mammalian expression vectors as indicated. The reporter plasmids (IFN-β-luciferase, PRDIII-I-luciferase, NF-κB-luciferase and pTK-Renilla) have been described previously [53,54]. SUMO-1, SUMO-3 constructs were kindly provided by Dr. Jinke Cheng (School of Medicine, Shanghai Jiao Tong University, Shanghai, China). The SENP7 siRNA-resistant form was generated with silent mutations introduced into the siRNA target sequence. All point mutations were generated by using a QuickChange XL site-directed mutagenesis method (Stratagene). All the plasmids were verified by sequencing. Recombinant GST-fusion proteins were purified from Escherichia coli (BL21) by using glutathione-Sepharose 4B resin (GE Healthcare, Piscataway, NJ).

### Manipulation of viruses and bacteria

HSV-1 and HSV-1-GFP were kindly provided by Dr. Wentao Qiao (Nankai University) and Dr. Chunfu Zheng (Suzhou University), respectively. HSV-1 was propagated and titered by plague assays on Vero Cells. Listeria monocytogenes (10403 serotype) was a gift from Dr. Youcun Qian (Institute of Health Sciences). Listeria monocytogenes was cultured in 3.7% Brain-Heart Infusion broth (BD Biosciences).

### Immunoprecipitation assay and immunoblot analysis

For immunoprecipitation assay, cells extracts were prepared by using RIPA buffer (50 mM Tris-HCl pH 7.4, 150 mM NaCl, 1 mM EDTA, 1% Triton X-100, 0.1% SDS, 0.5% deoxycholate) supplemented with a complete protease inhibitor cocktail (Roche), a PhosSTOP phosphatase inhibitor cocktail (Roche) and 20 mM N-ethylmaleimide (NEM). Lysates were incubated with the appropriate antibody for four hours to overnight at 4°C before adding protein A/G agarose for 2 hr. The immunoprecipitates were washed three times with the same buffer and eluted with SDS loading buffer by boiling for 5 min. For denaturing immunoprecipitation, cells were lysed in 1% SDS buffer (50 mM Tris-HCl pH 7.5, 150 mM NaCl, 1% SDS, 10 mM DTT) and boiled for thirty minutes. The lysates were centrifuged and diluted by 10-fold with Lysis buffer (50 mM Tris-HCl pH 7.5, 150 mM NaCl, 1 mM EDTA, 1% Triton X-100). The diluted lysates were immunoprecipitated with the indicated antibodies for four hours to overnight at 4°C before adding protein A/G agarose for 2 hr. After extensive wash, the immunoprecipitates were subjected to immunoblot analysis.

For immunoblot analysis, the samples were subjected to SDS-PAGE. The resolved proteins were then electrically transferred to a PVDF membrane (Millipore). Immunoblotting was probed with indicated antibodies. The protein bands were visualized by using a SuperSignal West Pico chemiluminescence ECL kit (Pierce). Signal intensities of immunoblot bands were quantified by Image J software.

### Luciferase reporter assays

Cells were transfected with reporter plasmids. Luciferase activity was assessed with a dual luciferase assay kit (Promega) and a Luminoskan Ascent luminometer (Thermo Scientific) [55].

### Antibodies and reagents

The polyclonal antibody against SENP7 was generated by immunizing rabbit with recombinant mouse SENP7 (716–1010 aa). The rabbit polyclonal antibody against cGAS was obtained by immunizing rabbit with recombinant mouse cGAS (362–643 aa). The antibodies against hemagglutinin (HA), Myc and IRF3 were purchased from Santa Cruz Biotechnology. Mouse monoclonal Flag antibody and β-actin antibodies were obtained from Sigma-Aldrich. The SUMO antibody was from Abcam. Phospho-IRF3 antibody, Phospho-TBK1 antibody, and rabbit DYKDDDDK tag antibody were from Cell Signaling Technology. TBK1 antibody was from Abcam. Anti-Flag (M2)-agarose and EZview red anti-HA affinity gel were from Sigma. Poly(dA:dT) was obtained from Sigma. Interferon stimulatory DNA (ISD) was prepared by annealing equimolar amounts of sense and antisense DNA oligonucleotides at 95°C for 10 min before cooling to room temperature. Oligonucleotides used are as follows: ISD (sense), 5′-TAC AGA TCT ACT AGT GAT CTA TGA CTG ATC TGT ACA TGA TCT ACA-3′; ISD (antisense), 5′-TGT AGA TCA TGT ACA GAT CAG TCA TAG ATC ACT AGT AGA TCT GTA-3′. cGAMP was from InvivoGen and was delivered into cultured cells by digitonin permeabilization method as previously described (Girardin et al., 2003).

### Real-time RT-PCR

Total RNA was isolated from indicated cells by using TRIzol reagent (Invitrogen) according to the manufacturer’s instructions, and then subjected to reverse transcription. The quantifications of gene transcripts were performed by real-time PCR using Power SYBR GREEN PCR MASTER MIX (ABI). GAPDH served as an internal control. PCR primers used to amplify the target genes are shown as follows: *Gapdh*: sense (5′-GAA GGG CTC ATG ACC ACA GT-3′), antisense (5′-GGA TGC AGG GAT GAT GTT CT-3′); *Ifnb*: sense (5′-AGA TCA ACC TCA CCT ACA GG-3′), antisense (5′-TCA GAA ACA CTG TCT GCT GG-3′); *Ifna4*: sense (5′-ACC CAC AGC CCA GAG AGT GAC C-3′), antisense (5′-AGG CCC TCT TGT TCC CGA GGT-3′); *Cxcl10*: sense (5′-CCT GCC CAC GTG TTG AGA T-3′), antisense (5′-TGA TGG TCT TAG ATT CCG GAT TC-3′); *OAS1*: sense (5′-GAA GGC AGC TCA CGA AAC-3′), antisense (5′-TTC TTA AAG CAT GGG TAA TTC-3′); *MX1*: sense (5′-GGG TAG CCA CTG GAC TGA-3′), antisense (5′-AGG TGG AGC GAT TCT GAG′); *ISG15*: sense (5′-TGT CGG TGT CAG AGC TGA AG-3′), antisense (5′-GCC CTT GTT ATT CCT CAC CA′).

### Measurement of cytokines

Concentrations of the cytokine in culture supernatants were measured by ELISA kit (R&D Systems) according to the manufacturer’s instructions.

### Native PAGE assay

Native gel electrophoresis for IRF3 dimerization was carried out as described previously [56].

### Immunostaining and confocal microscopy

Cells grown on coverslips were fixed for 15 min with 4% paraformaldehyde in PBS, permeabilized for 20 min in 0.1% Triton X-100 in PBS and blocked using 5% BSA for 1 hr. Then, the cells were stained with the indicated primary antibodies followed by incubation with fluorescent-conjugated secondary antibodies (Jackson ImmunoResearch). Nuclei were counterstained with DAPI (4,6-diamidino- 2-phenylindole) (Sigma-Aldrich). Slides were mounted using Aqua-Poly/ Mount (Dako). Images were captured using a Leica laser scanning confocal microscopy. The acquiring software was TCS (Leica, Solms, Germany).

### Ni-NTA pulldown analysis

For Ni-nitrilotriacetic acid resin (NTA) pulldown analysis, cells were lysed in His-Lysis Buffer (50 mM Tris-HCl pH 7.4, 300 mM NaCl, 1% Triton X-100, 20 mM imidazole, 10 mM β-ME) supplemented with 1 mM PMSF. After centrifugation, the supernatants were collected and incubated with 20 μL Ni-NTA agarose beads (Qiagen) for 4 hr at 4°C. After extensively washing with His-Lysis Buffer containing 20 mM imidazole, the precipitates were subjected to SDS-PAGE followed by immunoblot analysis or eluted with TBS containing 300 mM imidazole and subsequently subjected to biotin-pulldown analysis.

### *In vitro* SUMOylation assay

Purified His-Flag-cGAS, His-SUMO was mixed with E1 (50 nM) and E2 (0.3 μM) (Boston Biochem) in a reaction buffer containing 50 mM Tris-HCl, pH 7.5, 5 mM MgCl_2_, 2 mM ATP. The reaction was carried out at 37°C for 120 min and then resolved by SDS-PAGE. SUMOylated products were detected by immunoblotting with indicated antibodies.

### cGAS enzymatic activity assay

A total of 10 μM purified cGAS or mutant variants were incubated with the indicated DNA and reaction buffer (20 mM HEPES, pH 7.5, 5 mM MgCl_2_, 2 mM ATP, 2 mM GTP) at 37°C for 2 hr. Samples were centrifuged at 16,000 g for 10 min. The product in the supernatant was separated from cGAS and DNA by ultrafiltration. The samples were diluted by 5-fold and analyzed on a MonoQ ion exchange column (GE Healthcare) equilibrated with the running buffer (50 mM Tris-HCl pH 8.5) and eluted with a NaCl gradient of 0 to 0.5 M in the running buffer.

### FRET assay

The plasmids GFP-cGAS /mCherry-cGAS, which express GFP- and mCherry-tagged proteins at a ratio of 1:1, were transfected into HeLa cells. To calculate the apparent efficiency of FRET, we used the following two spectra obtained during the process of generating the FRET emission spectrum. The GFP-emission spectrum was obtained once before and once after photobleaching cherry. Each data set was based on >20 individual cells. FRET efficiency was calculated with the following formula: FRET% = (GFP after bleaching − GFP before bleaching) / GFP after bleaching) × 100. Statistical significance was determined with an unpaired t-test.

### Histological analysis

Tissues were fixed in 4% paraformaldehyde, embedded in paraffin, cut into sections, and placed on adhesion microscope slides. Sections were subjected to immunohistochemical (IHC) staining according to standard procedures. The anti-HSV-1 antibody (Abcam) was used for staining.

### Statistics

Student’s t test was used for the statistical analysis of two independent treatments. Mouse survival curves and statistics were analyzed using the Mantel-Cox Log-rank test. A non-parametric Mann-Whitney test was used to compare the gene expression in SLE patients and normal healthy subjects. Non-parametric correlation test (Spearman's rank correlation coefficient) were used to measure the degree of association between the expression of *SENP7* and IFN score. For all tests, a P value of < 0.05 was considered statistically significant.

## Supporting Information

S1 Fig (Related to Fig 1). SENP7 deficiency impairs intracellular DNA-mediated type I interferon production. (**A**) mRNA levels of SENP7, SENP1 and SENP3 in peripheral blood samples from healthy donors (n = 28) and SLE patients (n = 27). As for SENP7 comparison, *P* < 0.0001 (Mann-Whitney test). Each symbol represents an individual subject; horizontal lines indicate the mean. (**B**) SENP7 mRNA level versus IFN-inducible genes mRNA level (IFN score) in peripheral blood samples from SLE patients. Each symbol represents an individual subject. *r* = 0.4151; *P* = 0.0313 (Spearman’s rank correlation test). (**C**) MEFs were transfected with the negative control (N.C.) or *Senp7* siRNAs (upper panel). HEK293T cells were transfected with Flag-tagged SENP7, and then treated with the nonspecific control (N.C.) or *Senp7* siRNAs (lower panel). Cell lysates were immunoblotted with the indicated antibodies. (**D**) MEFs transfected with the indicated siRNAs were stimulated with ISD. Induction of *Ifnb*, *Ifna4* and *Cxcl10* mRNAs was measured by quantitative PCR. (**E, F**) SENP7-deficient MEFs were transfected with the indicated SENP7 constructs. After ISD stimulation, induction of *Ifnb* and *Ifna4* mRNA was measured by quantitative PCR (**E**). And the supernatants were collected and assayed for IFN-β and IFN-α4 production by ELISA (**F**). Graphs show the mean ± s.d. of triplicates and data shown are representative of three independent experiments. Statistical differences are calculated compared to untreated control samples. n.s., not significant, **P < 0.01 (two-tailed t-test).
(TIF)

S2 Fig (Related to Fig 4). SENP7 interacts with cGAS. (**A**) MEFs transfected with the indicated siRNAs were stimulated with cGAMP. Induction of *Ifnb*, *Ifna4* and *Cxcl10* mRNAs was measured by quantitative PCR. (**B**) MEFs transfected with the indicated siRNAs were treated or not with Vesicular stomatitis virus (VSV) for various time periods, and cell extracts were analyzed for TBK1/IRF3 phosphorylation and IRF3 dimerization by SDS-PAGE and native PAGE, respectively. (**C**)HEK293T cells were transfected with the indicated plasmids. 24 hr after transfection, cell lysates were immunoprecipitated with an anti-Myc antibody or normal IgG, and then immunoblotted with the indicated antibodies. (**D**) Flag-tagged SENP7 or its truncations were individually transfected into cells before the cell lysates were immunoprecipitated with Flag-beads and then immunoblotted with the indicated antibodies. (**E**) HA-tagged cGAS or its truncations were individually transfected into HEK293T cells along with Flag-tagged SENP7. The cell lysates were immunoprecipitated with Flag-beads and then immunoblotted with the indicated antibodies. Graphs show the mean ± s.d. of triplicates and data shown are representative of three independent experiments. Statistical differences are calculated compared to untreated control samples. n.s., not significant (two-tailed t-test).
(TIF)

S3 Fig (Related to Fig 4). Lysine 335, 372, 382 of cGAS are the major SUMOylation sites on cGAS. (A) Flag-tagged mouse cGAS or its mutants were individually transfected into HEK293T cells along with HA-tagged SUMO-2/3. Cell lysates were subjected to a two-step immunoprecipitation, and then immunoblotted with the indicated antibodies. (B,C) HEK293T cells were transfected with Flag-tagged mouse cGAS or its mutants along with HA-tagged SUMO-2/3. Cell lysates were subjected to a two-step immunoprecipitation, and then immunoblotted with the indicated antibodies. K3R denotes cGAS with lysine residues 335/ 372/ 382 mutated to arginine.
(TIF)

S4 Fig (Related to Fig 5). Lysine 335, 372, 382 of cGAS are critical for its function. (**A**) Schematic representation of cGAS orthologs. (**B**) HEK293T cells were transfected with the indicated plasmids. 24 hr after transfection, cell lysates were incubatd with biotin-ISD before streptavidin-conjugated beads was added. DNA-binding activity of cGAS was assessed by immunoblot analysis of the ISD precipitates and total lysates (below) with the indicated antibody. (**C**) HEK293T cells were transfected with STING and cGAS WT/K3A plasmids together with the IFN-β promoter reporter and pTK-Renilla reporter plasmids. 24 hr post-transfection, luciferase assays were performed. All data shown are representative of three independent experiments.
(TIF)

S5 Fig Knockdown of SENP7 impairs intracellular DNA-mediated type I interferon production in L929s and primary BMDMs. (A, B) L929 cells (A) or BMDMs (B) transfected with the indicated siRNAs were infected with HSV-1. Induction of *Ifnb*, *Ifna4* and *Cxcl10* mRNAs was measured by quantitative PCR. Graphs show the mean ± s.d. and data shown are representative of three independent experiments. *P < 0.05; **P < 0.01 (two-tailed t-test).
(TIF)

S6 Fig SENP7 is dispensable for innate immune defense against RNA virus invasion. (A) MEFs transfected with the nonspecific control (N.C.) or *SENP7* siRNAs were treated or not with NDV (Newcastle disease virus). Equal volumes of culture supernatants from these treatments were applied to fresh MEF cells, followed by HSV-1 infection. The proliferation of cells was examined by crystal violet staining. Scale bars represent 200 μm. (B) MEFs transfected with the nonspecific control (N.C.) or *SENP7* siRNAs were infected with NDV. The titers of NDV were determined by standard plaque assay. (C) NDV-GFP replication in MEFs transfected with the indicated siRNAs was visualized by fluorescence microscopy. Graphs show the mean ± s.d. and data shown are representative of three independent experiments. Statistical differences are calculated compared to untreated control samples. n.s., not significant (two-tailed t-test).
(TIF)
