# Supplementary figures and images for: SENP7 Potentiates cGAS Activation by Relieving SUMO-Mediated Inhibition of Cytosolic DNA Sensing
Source: PLoS Pathog. 2017 Jan 17;13(1):e1006156. doi: 10.1371/journal.ppat.1006156 (PMC5271409; doi:10.1371/journal.ppat.1006156)

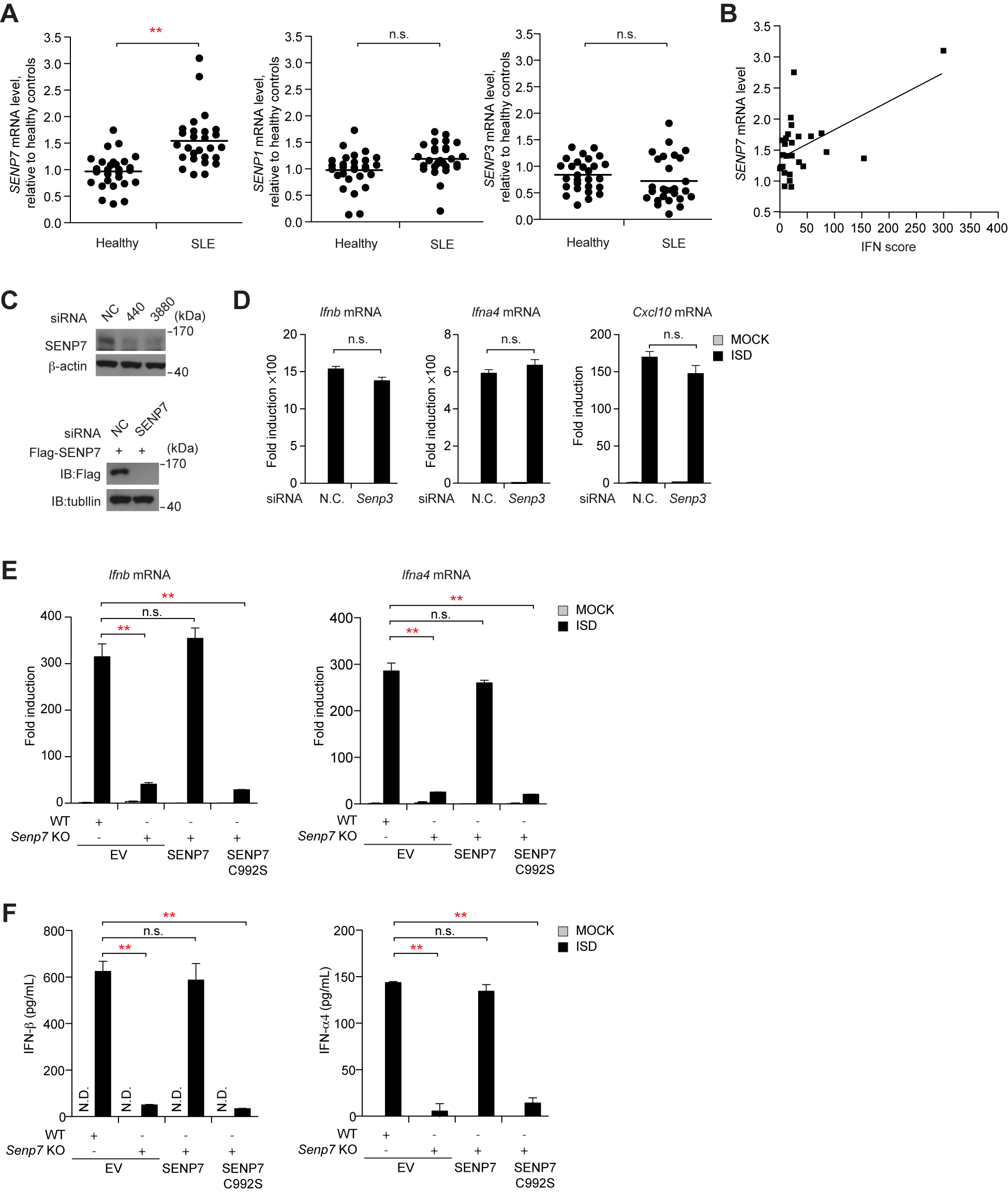

Supplement: S1 Fig — (A) mRNA levels of SENP7, SENP1 and SENP3 in peripheral blood samples from healthy donors (n = 28) and SLE patients (n = 27). As for SENP7 comparison, P < 0.0001 (Mann-Whitney test). Each symbol represents an individual subject; horizontal lines indicate the mean. (B) SENP7 mRNA level versus IFN-inducible genes mRNA level (IFN score) in peripheral blood samples from SLE patients. Each symbol represents an individual subject. r = 0.4151; P = 0.0313 (Spearman’s rank correlation test). (C) MEFs were transfected with the negative control (N.C.) or Senp7 siRNAs (upper panel). HEK293T cells were transfected with Flag-tagged SENP7, and then treated with the nonspecific control (N.C.) or Senp7 siRNAs (lower panel). Cell lysates were immunoblotted with the indicated antibodies. (D) MEFs transfected with the indicated siRNAs were stimulated with ISD. Induction of Ifnb, Ifna4 and Cxcl10 mRNAs was measured by quantitative PCR. (E, F) SENP7-deficient MEFs were transfected with the indicated SENP7 constructs. After ISD stimulation, induction of Ifnb and Ifna4 mRNA was measured by quantitative PCR (E). And the supernatants were collected and assayed for IFN-β and IFN-α4 production by ELISA (F). Graphs show the mean ± s.d. of triplicates and data shown are representative of three independent experiments. Statistical differences are calculated compared to untreated control samples. n.s., not significant, **P < 0.01 (two-tailed t-test). (TIF) [file ppat.1006156.s001.tif]

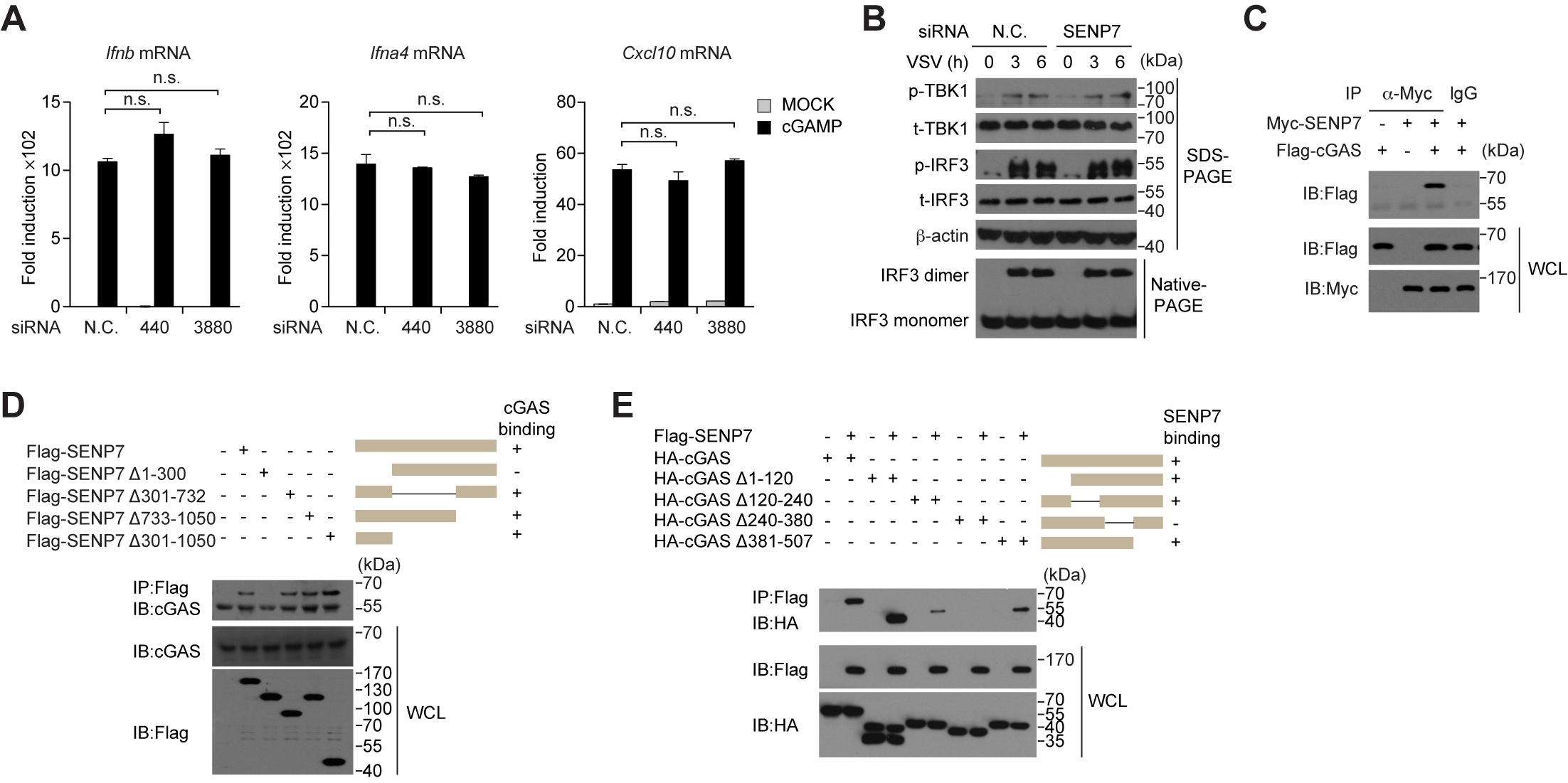

Supplement: S2 Fig — (A) MEFs transfected with the indicated siRNAs were stimulated with cGAMP. Induction of Ifnb, Ifna4 and Cxcl10 mRNAs was measured by quantitative PCR. (B) MEFs transfected with the indicated siRNAs were treated or not with Vesicular stomatitis virus (VSV) for various time periods, and cell extracts were analyzed for TBK1/IRF3 phosphorylation and IRF3 dimerization by SDS-PAGE and native PAGE, respectively. (C)HEK293T cells were transfected with the indicated plasmids. 24 hr after transfection, cell lysates were immunoprecipitated with an anti-Myc antibody or normal IgG, and then immunoblotted with the indicated antibodies. (D) Flag-tagged SENP7 or its truncations were individually transfected into cells before the cell lysates were immunoprecipitated with Flag-beads and then immunoblotted with the indicated antibodies. (E) HA-tagged cGAS or its truncations were individually transfected into HEK293T cells along with Flag-tagged SENP7. The cell lysates were immunoprecipitated with Flag-beads and then immunoblotted with the indicated antibodies. Graphs show the mean ± s.d. of triplicates and data shown are representative of three independent experiments. Statistical differences are calculated compared to untreated control samples. n.s., not significant (two-tailed t-test). (TIF) [file ppat.1006156.s002.tif]

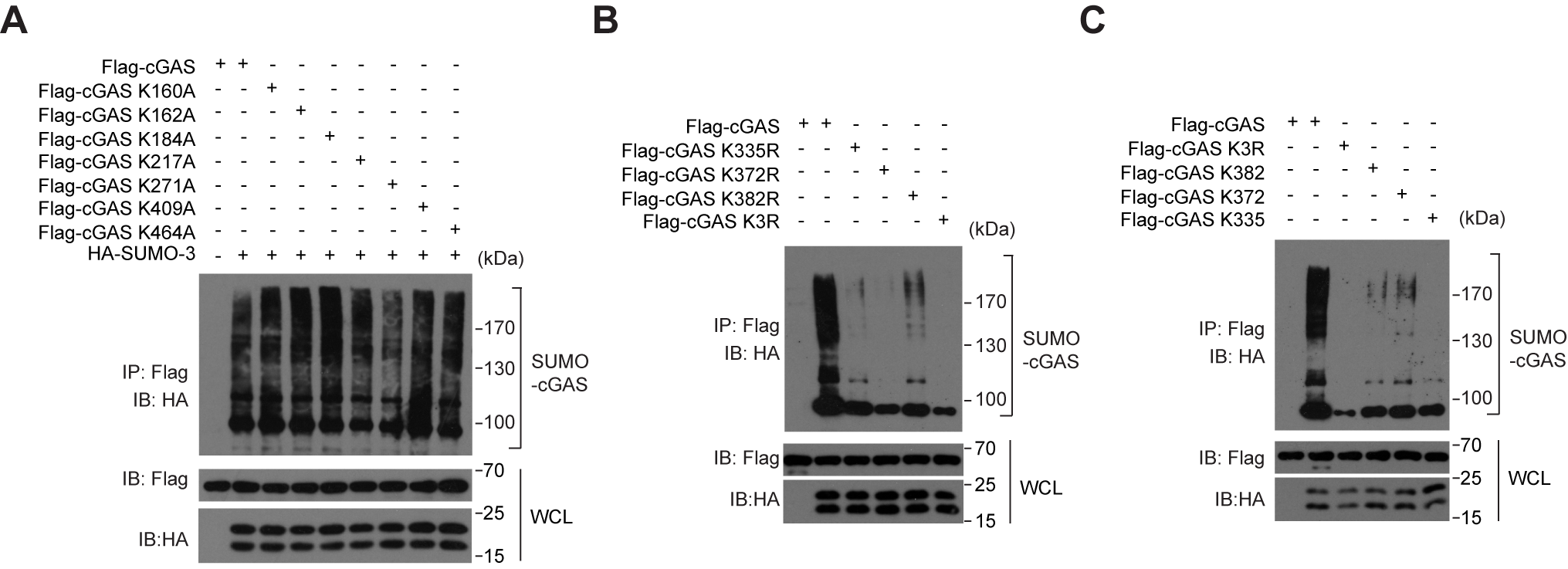

Supplement: S3 Fig — (A) Flag-tagged mouse cGAS or its mutants were individually transfected into HEK293T cells along with HA-tagged SUMO-2/3. Cell lysates were subjected to a two-step immunoprecipitation, and then immunoblotted with the indicated antibodies. (B,C) HEK293T cells were transfected with Flag-tagged mouse cGAS or its mutants along with HA-tagged SUMO-2/3. Cell lysates were subjected to a two-step immunoprecipitation, and then immunoblotted with the indicated antibodies. K3R denotes cGAS with lysine residues 335/ 372/ 382 mutated to arginine. (TIF) [file ppat.1006156.s003.tif]

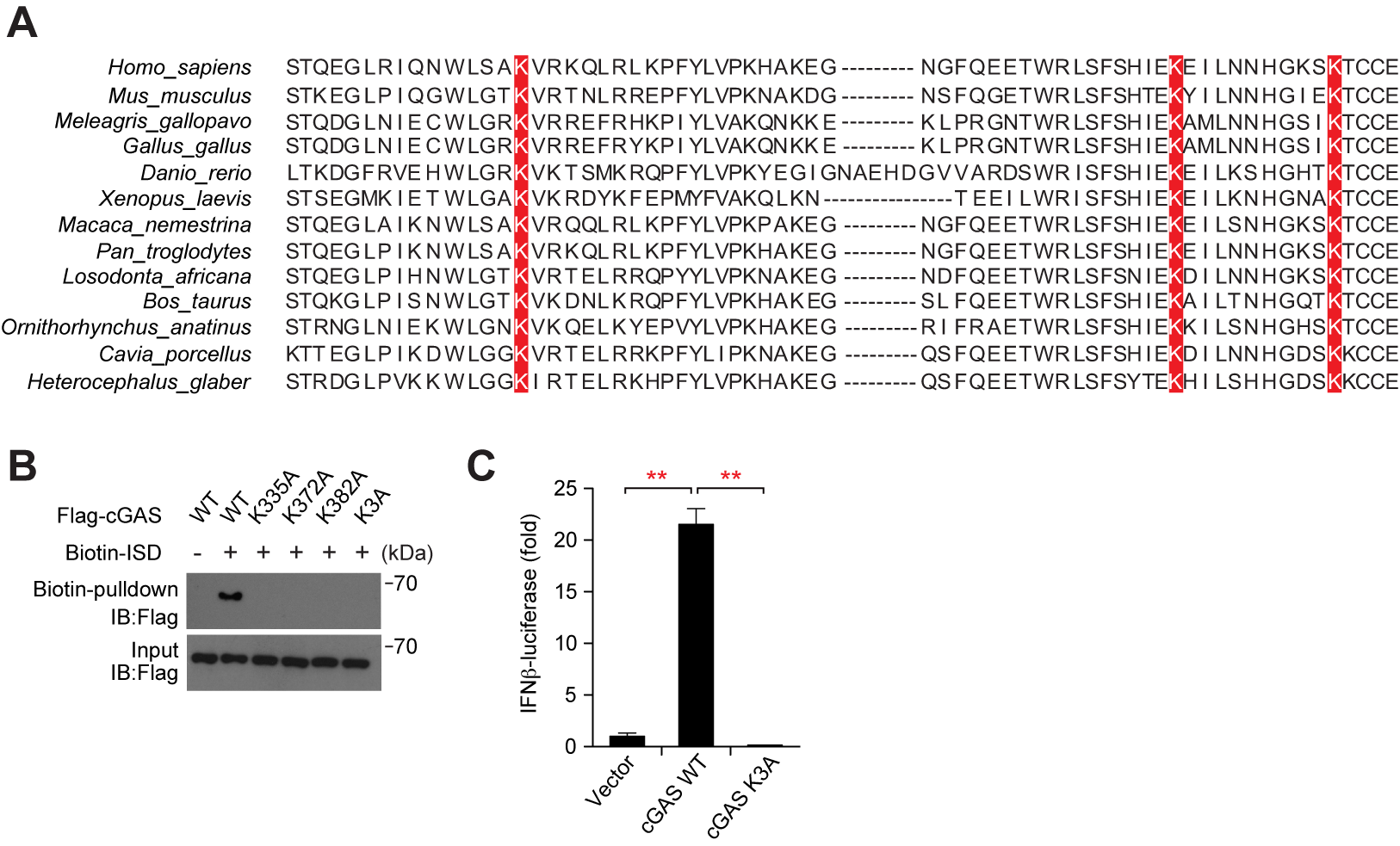

Supplement: S4 Fig — (A) Schematic representation of cGAS orthologs. (B) HEK293T cells were transfected with the indicated plasmids. 24 hr after transfection, cell lysates were incubatd with biotin-ISD before streptavidin-conjugated beads was added. DNA-binding activity of cGAS was assessed by immunoblot analysis of the ISD precipitates and total lysates (below) with the indicated antibody. (C) HEK293T cells were transfected with STING and cGAS WT/K3A plasmids together with the IFN-β promoter reporter and pTK-Renilla reporter plasmids. 24 hr post-transfection, luciferase assays were performed. All data shown are representative of three independent experiments. (TIF) [file ppat.1006156.s004.tif]

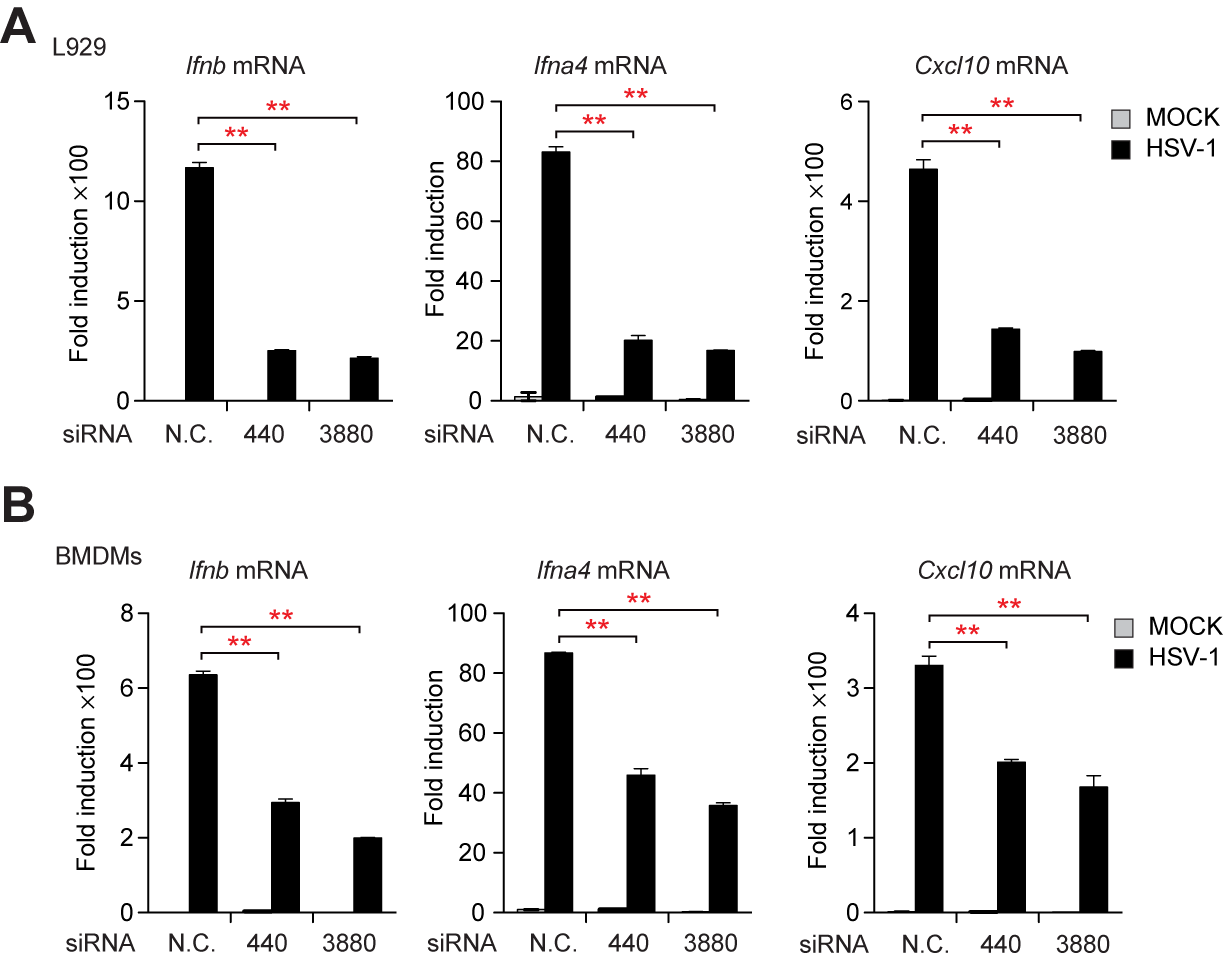

Supplement: S5 Fig — (A, B) L929 cells (A) or BMDMs (B) transfected with the indicated siRNAs were infected with HSV-1. Induction of Ifnb, Ifna4 and Cxcl10 mRNAs was measured by quantitative PCR. Graphs show the mean ± s.d. and data shown are representative of three independent experiments. *P < 0.05; **P < 0.01 (two-tailed t-test). (TIF) [file ppat.1006156.s005.tif]

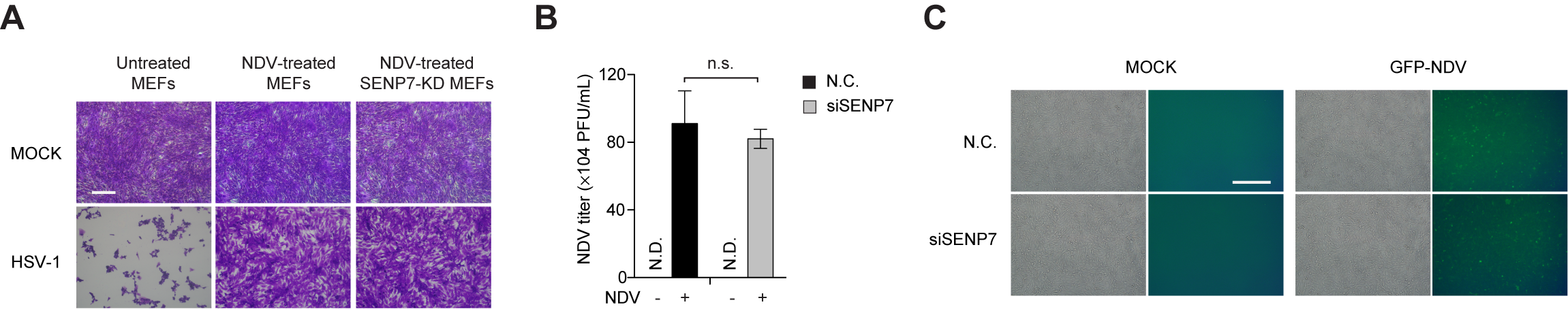

Supplement: S6 Fig — (A) MEFs transfected with the nonspecific control (N.C.) or SENP7 siRNAs were treated or not with NDV (Newcastle disease virus). Equal volumes of culture supernatants from these treatments were applied to fresh MEF cells, followed by HSV-1 infection. The proliferation of cells was examined by crystal violet staining. Scale bars represent 200 μm. (B) MEFs transfected with the nonspecific control (N.C.) or SENP7 siRNAs were infected with NDV. The titers of NDV were determined by standard plaque assay. (C) NDV-GFP replication in MEFs transfected with the indicated siRNAs was visualized by fluorescence microscopy. Graphs show the mean ± s.d. and data shown are representative of three independent experiments. Statistical differences are calculated compared to untreated control samples. n.s., not significant (two-tailed t-test). (TIF) [file ppat.1006156.s006.tif]
